# TraceBERT—A Feasibility Study on Reconstructing Spatial–Temporal Gaps from Incomplete Motion Trajectories via BERT Training Process on Discrete Location Sequences

**DOI:** 10.3390/s22041682

**Published:** 2022-02-21

**Authors:** Alessandro Crivellari, Bernd Resch, Yuhui Shi

**Affiliations:** 1 Department of Computer Science and Engineering, Southern University of Science and Technology, Shenzhen 518055, China; crivellari@sustech.edu.cn; 2 Department of Geoinformatics—Z_GIS, University of Salzburg, 5020 Salzburg, Austria; 3 Center for Geographic Analysis, Harvard University, Cambridge, MA 02138, USA

**Keywords:** BERT, neural networks, trajectories, human mobility, spatial–temporal gaps

## Abstract

Trajectory data represent an essential source of information on travel behaviors and human mobility patterns, assuming a central role in a wide range of services related to transportation planning, personalized recommendation strategies, and resource management plans. The main issue when dealing with trajectory recordings, however, is characterized by temporary losses in the data collection, causing possible spatial–temporal gaps and missing trajectory segments. This is especially critical in those use cases based on non-repetitive individual motion traces, when the user’s missing information cannot be directly reconstructed due to the absence of historical individual repetitive routes. Inserted in the context of location-based trajectory modeling, we tackle the problem by proposing a technical parallelism with the natural language processing domain. Specifically, we introduce the use of the Bidirectional Encoder Representations from Transformers (BERT), a state-of-the-art language representation model, into the trajectory processing research field. By training deep bidirectional representations from unlabeled location sequences, jointly conditioned on both left and right context, we derive an explicit predicted estimation of the missing locations along the trace. The proposed framework, named TraceBERT, was tested on a real-world large-scale trajectory dataset of short-term tourists, exploring an effective attempt of adapting advanced language modeling approaches into mobility-based applications and demonstrating a prominent potential on trajectory reconstruction over traditional statistical approaches.

## 1. Introduction

The research interest on human mobility analysis has extensively expanded over the last few years, driven by the increasing availability of trajectory data acquired by pervasive motion tracking technologies. These data represent a primary source of information on human travel behaviors [1,2], giving rise to a multitude of data mining investigations on motion analysis and trajectory-related applications [3,4,5,6], ranging from personalized recommendation systems [7,8], to transportation planning [9,10], to resource management plans [11,12]. In today’s digital world of location-based services and positioning devices, the collection of mobility data covers a variety of acquisition modalities, including mobile phone networks, GPS signals, and social media platforms. The resulting tracking of large numbers of people leads to the creation of big datasets of historical motion traces, whose use has been widely explored according to several different tasks, such as trajectory prediction [13,14,15], trajectory classification [16,17], motion flow modeling [18,19,20], or activity recognition [21,22].

When dealing with this kind of mobility data, however, the primary issue is represented by the fact that their quality is rarely optimal, presenting, in most cases, a lack of completeness and a certain degree of information loss [23,24]. Trajectory recordings are indeed often characterized by temporary losses in the data collection, causing possible spatial–temporal gaps and missing trajectory segments [25,26,27]. These losses can be of various nature, namely depending on event-based recording modalities, ad hoc acquisition strategies, signal interferences, or technical malfunctioning [28,29,30]. Our research aims to find an effective way to properly fill these geospatial information gaps.

The research task is therefore interpreted as inferring the missing spatial–temporal observations of an individual, based on the known visited locations along the trajectory. While daily life movements can generally be easily reconstructed, due to the repetitive nature of the mobility routine, a critical condition is represented by cases of non-repetitive behaviors, whereby a motion trace lacks spatial and temporal regularity. In that condition, the user’s missing information cannot be directly inferred from a sequential approximation of a single probability distribution, because of the absence of historical individual repetitive routes. Our paper intentionally targets this specific situation.

Inserted into the context of location-based trajectory modeling, we tackle the problem by proposing a technical parallelism with the natural language processing (NLP) domain. Specifically, we present an original approach called TraceBERT, introducing the use of the Bidirectional Encoder Representations from Transformers (BERT) [31], a state-of-the-art language representation model, into the trajectory processing field.

The way that NLP developed its powerful methodologies represents a hotbed of analytical tools for sequential-based problems. From a technical perspective, the processing of text in the form of sequences of words can be generalized into a sequential processing of generic categorical entities. Among many disciplines, geospatial and urban studies have also taken inspiration from the NLP world; examples include the adoption of neural embeddings to model locations, points of interest and functional areas [32,33,34,35,36], and the use of advanced deep learning algorithms in the context of trajectory analysis [37,38,39].

**Figure 1:**
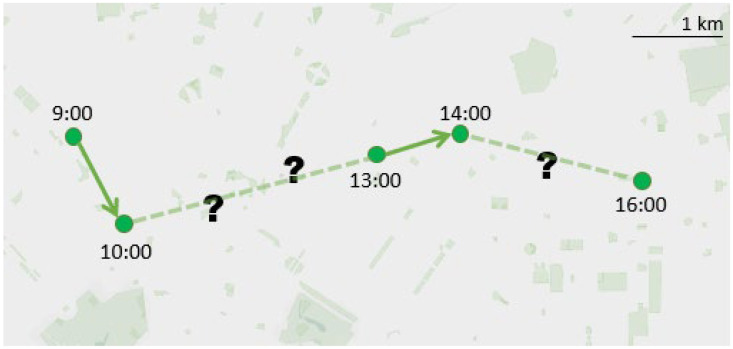
Trajectory reconstruction problem: predict the missing locations given the known recorded locations along the trace.

Our work pushes this modeling parallelism to a further level, transforming the state-of-the-art language representation network into a trajectory reconstruction model. Inspired by the masked-language modeling (MLM) approach [31], which masks certain words over the text and attempts to re-identify them based on the context provided by the non-masked words, we aim to predict the missing locations along the trajectory by leveraging the context provided by the known recorded locations in the sequence, as reported in Figure 1.

The underlying idea is to apply bidirectional training of the transformer architecture, a popular attention-based neural network model [40], to location-based trajectory representations, making sense of the complete information on mobility context and flow along each motion trace. By training deep bidirectional representations from unlabeled location sequences, jointly conditioned on both the left and right context, we derive an explicit predicted estimation of the missing locations along the trace. The methodological procedure consists of four steps: first, raw traces are pre-processed into discrete location sequences; then, the training set is defined by randomly hiding a portion of locations in each trajectory, replacing their unique identifiers with a masking token in the corresponding position along the sequence; subsequently, the BERT model is trained through backpropagation, by feeding the partially hidden location sequences with the goal of optimizing the correct prediction in the masked positions; finally, the model is evaluated by means of testing trajectories, leading to an automatic gap-filling of the missing locations along each trace. The model is intended to capture motion patterns directly from the collective processing of location sequences, without requiring any manual feature extraction or external information.

The proposed framework was tested on a real-world large-scale trajectory dataset of short-term tourists. In contrast to daily life’s mobility, implying a significant probability of returning to a limited number of highly frequented locations [41,42], the natural characterization of tourists’ motion behavior is made of non-repetitive trajectories of users moving in unfamiliar areas. Moreover, the focus on large-scale movements entails a wide territory, determining further issues such as trajectory sparseness and a multitude of locations. Experiments demonstrated the effectiveness of our proposed deep learning approach, reporting a higher feasibility trait when compared to traditional statistical methods in this mobility regime. By defining a valid system to disclose missing spatial–temporal information in movement data recordings, TraceBERT arises as a novel beneficial trajectory-based application of adapted NLP-inspired advanced neural network models within a geospatial discipline.

## 2. Methodology

The process is designed for automatic detection of hidden patterns from collective historical human motion data, in order to reconstruct a complete version of individual users’ incomplete input trajectories. The task is formally defined as follows: Given an individual user’s trajectory, sampled at a given time step, affected by spatial information gaps in correspondence of some specific time spans, our modeling solution allows filling of the gaps by inferring the unknown visited locations at those points in time.

The methodological details are organized into four subsections. The structural steps are the following:

- Trajectory pre-processing, defining the procedure of transforming the original raw trajectory recordings, continuous in time and space, into discrete location sequences;
- Location masking, reporting how the space–time information gaps are artificially created by masking a portion of elements in the sequences;
- BERT model training, describing how the derived incomplete traces are processed by the deep learning model, allowing the system to learn the underlying semantics of user mobility patterns;
- Location gap inference, characterizing the evaluation phase as an automatic generation of location data in correspondence of missing trajectory segments, turning incomplete input traces into complete output sequences.

### 2.1. Trajectory Pre-Processing

The first methodological step is represented by a process of trajectory discretization, conforming raw traces to an adequate input format for the neural network model. A raw trajectory recording is a series of chronologically ordered track points, carrying information on the geographic coordinates and time stamp of acquisition, namely $T=\{p_{i}|i=1,2,3,\ldots ,N\}$, where $p_{i}=\left(lon_{i},lat_{i},t_{i}\right)$. The discretization task involves transforming the continuous longitude and latitude values into discrete locations and the continuity of time into fixed time steps.

The pre-processed trajectory representation is intended to be described as a sequence of location identifiers $T=\left[loc\_ID_{t},\;loc\_ID_{2t},\;loc\_ID_{3t},\ldots \right]$, referring to fixed consecutive time steps of duration t (e.g., if t=1 h, the sequence is based on the concatenation of user’s positions at each consecutive hour). In general, if more than one record was acquired within the same time step, the chosen location is identified as the one associated to the majority of track points in that time span. The length of the fixed time unit is case specific, conditioned by the data source and the desired set up. A long unit may negatively affect the study of fine resolution movements; a short unit may critically fragment trajectories in the case of discontinuous traces. Space resolution is also-case specific, allowing for a higher- or lower-level discretization depending on the data sparseness and the planned configuration. Moreover, when human mobility is not uniformly distributed across the territory, inaccessible locations may be discarded, avoiding worthless computational effort.

In conclusion, a user’s pre-processed trajectory is a sequence of discrete identifiers unfolding in fixed time steps, each of them representing a specific unique location within a finite set of possible reference locations over the territory.

### 2.2. Location Masking

To enable the definition of the final data format for the BERT training, a process of location masking is included. Given the assumption that the inference goal is to fill the gaps in missing trajectory segments, the location masking procedure aims indeed to artificially generate location gaps, so that the model can be trained on learning how to fill them.

Considering a certain sequence $\left[LOC846,LOC37,LOC911,LOC51,LOC89,\ldots \right]$, the idea is to randomly mask, with a defined masking probability, some of the locations along the sequence, therefore potentially transforming it into a corresponding masked version $\left[LOC846,\langle masked\rangle ,LOC911,LOC51,\langle masked\rangle ,\ldots \right]$. Each trajectory undergoes this masking process; the model training relies on feeding masked trajectories as an input, and their corresponding hidden locations as a desired output, with the goal of learning how to perform meaningful trajectory reconstructions from a spatial–temporal perspective. The model is intended to be trained by optimizing the probability of guessing the artificially masked locations correctly, with help of the contextual information provided by the non-masked locations.

In other words, we aim to reconstruct the complete trajectory based on an incomplete input, by predicting the masked locations along the input sequence. The intrinsic prediction task assumes the meaning of generating a reasonable path for the unknown trajectory segments, based on the known related contextual spatial–temporal information.

### 2.3. BERT Model Training

To perform the sequence reconstruction process, we adapted the MLM training approach featuring BERT [31], current state-of-the-art in most language processing tasks. While we leverage the same internal architecture and characteristic training process, the model is, in fact, trained from scratch on the previously described location-based pre-processed trajectories. Conceptually, the original implementation, conceived for dealing with sequences of words (sentences), is adapted into a processing of location sequences (motion traces).

**Figure 2:**
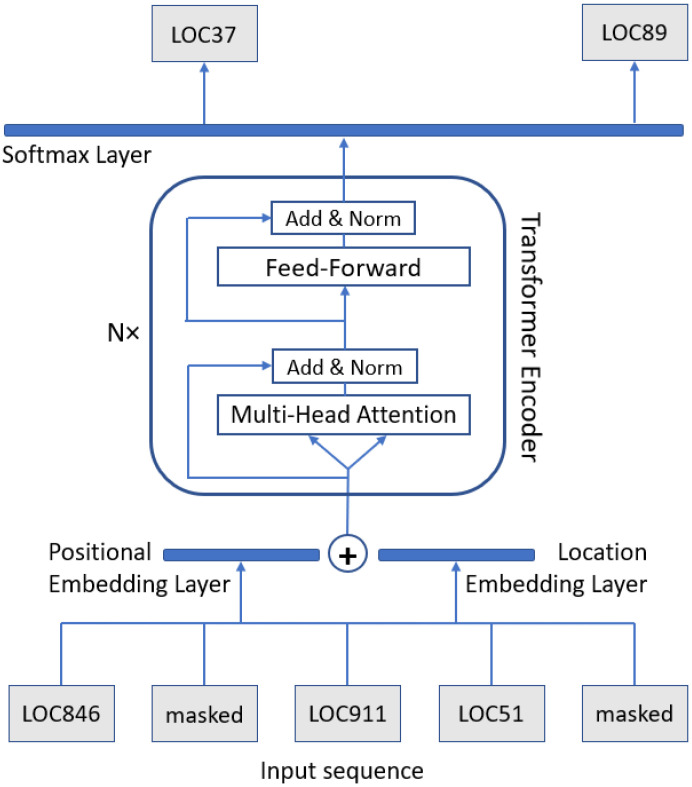
Visual representation of the TraceBERT model architecture.

An exemplifying representation is depicted in Figure 2. MLM consists of feeding BERT with a partially masked sequence, and consequently optimizing its weights for properly revealing, as an output, the masked elements of such sequence. The BERT architecture allows performing bidirectional learning, inferring the context of each element along the sequence by observing the elements appearing both before and after it (in contrast to previous methodologies using unidirectional predictions [43], or a combination of left-to-right and right-to-left training to approximate bidirectionality [44]). Therefore, our model uses the full context in the trace to predict the masked location, taking both the previous and next locations into account at the same time. Analogously to the original BERT, which learns linguistic patterns through contextual word occurrences along the sentences, our TraceBERT aims to model motion patterns by processing location visits along individual mobility paths.

The BERT model design is based on stacking multiple transformer encoders on top of each other. The transformer architecture refers to the multi-head attention module that has shown substantial success in many vision and language tasks [40,45,46]. Each transformer encoder consists of two layers: a multi-head self-attention layer, and a position-wise fully connected feed-forward network. The attention layer encodes each element’s relations with every other element in the sequence, giving more importance to the most relevant ones; the feed-forward network then applies itself to each resulting element’s output vector parallelly. Overall, in our case, the process consists of determining the contextual relations between the locations in the trace, assessing their relevance and acquiring “semantic” information. Instead of looping multiple times over the input (as in the case of recurrent neural networks [47]), BERT uses multiple attention layers through which the information passes linearly. To address ordering issues, the transformer architecture encodes the position of a location along the sequence directly into a dedicated embedding vector, as a marker for attention layers. Indeed, in addition to the traditional entity embedding input representation as low-dimensional vectors of location identifiers, the further use of positional embeddings is provided. Since the multi-head attention layers are time-distributed (the output has a one-to-one correspondence with the input at the same index), they do not directly grasp the relative order of the elements in the sequence, but they only look at their relations; therefore, external positioning information is required to be added. Finally, the model includes also skip-level residual connections, to help information traverse in case of deep networks.

By adding a fully connected softmax layer on top of the final encoder output vector, the prediction probability distribution of the masked location is computed over the totality of locations in the “vocabulary”: an input masked location may be predicted, for instance, as LOC37 with a probability of 40%, as LOC55 with a probability of 10%, as LOC89 with a probability of 5%, etc.; the location with the highest probability represents the first choice of the output location. The probability-based outcome reshapes the problem into a regular classification task, allowing for the use of the cross-entropy loss function between the output probability distribution and the real label. The loss is calculated only over the masked locations, so that the model learns to predict locations it has not seen, while observing the context around them. The process relies on backpropagation and mini-batch stochastic training to determine the required gradient changes and the resulting weight optimizations.

### 2.4. Location Gap Inference

The inference phase refers to the generation and evaluation of the results, assessing the generalization capabilities of the model, after the training process is concluded and optimized weights are assigned. The underlying idea is to feed new location sequences, unseen by the model during training, and explore the outcomes that the model provides. The generation of missing locations is therefore solely based on a collection of new incomplete input trajectories and the same parameter configuration defined at the end of the training phase. Given an input sequence with location gaps, TraceBERT generates each missing location as a function of its position along the sequence and the contextual known locations preceding and following it, defining a plausible trajectory path that could have been traveled by a visitor based on the initially provided partial information.

For instance, given an input sequence $\left[LOC83,\langle unknown\rangle ,LOC92,LOC721,LOC87,\ldots \right]$, the goal is to reveal the unknown location based on the information derived from the known ones, hence taking the whole known context into account. LOC83 and LOC92 may suggest that the unknown location is placed in a geographic area between them, but this may comprise many candidate locations; the further conditions provided by the presence of LOC721 and LOC87 narrow down the search, identifying the most likely missing location (or a small pool of most likely candidates). While for humans this would require a deep study on the complexity of motion activities, for BERT it just comes from having observed a lot of trajectories and learned their collective motion patterns. The model may not know the functional characteristics of LOC83, LOC92, LOC721, and LOC87, but it does find an answer based on the learned mobility correspondences and location co-occurrences. The outcome of this process relies on an advanced automatic comprehension of the underlying sequential motion patterns across the territory.

## 3. Experiment

The model was implemented and executed on TensorFlow, using AWS EC2 p3.2xlarge GPU instance.

### 3.1. Dataset

We evaluated the TraceBERT framework on non-repetitive motion trajectories of short-term visitors in a foreign country. In particular, we leveraged a real-world large-scale collection of seven months of anonymized mobile phone call detail records (CDRs) of roamers in Italy, whose mobility traces cover the study area with redundancies, creating a sufficiently large and complete dataset. To fall in the context of individual short traces and non-repetitive behaviors, we only selected those visitors located in the country for a maximum of two weeks; moreover, we discarded the completely stationary users. From a data acquisition perspective, each user’s geographic information was recorded according to the position of the device associated to any mobile phone activity, registering the coverage area of the principal antenna and the corresponding time stamp. CDR data have been extensively used in human mobility research and trajectory-related studies [48,49,50,51].

To overcome the erratic profile of mobile activity and address the purpose of modeling large-scale movements, we pre-processed mobility traces into sequences unfolded in 1 h time step, with a minimum spatial resolution of 2 km. Accordingly, the reference points over the territory were selected as the antennas counting the highest number of connections within the minimum spatial resolution, consequently merging the other coordinates to the closest reference point; if more than one recording was acquired within the same hour, the current location of the user was chosen as the one identified by the majority of those recordings. Very rare locations, almost randomly visited, were discarded, not being significant to the overall trend of visitors’ travel behavior. In any case, different selections of time and space resolution are possible, based on the targeted application and the data characteristics.

Our final dataset consists of hourly location sequences, comprising a total of 5903 possible discrete geographic points over the territory. To appropriately align different acquisition profiles on the focus for short mobility behaviors and make prediction results comparable over the entire dataset, we proceeded to divide trajectories into segments of a standard length of 7 h, determining 13 million consistent trajectory segments (with a median displacement of 36.1 km) generated by a total of 1.4 million users. We consider this large amount of data as an acceptable representative approximation of the real large-scale motion activity of short-term foreign visitors.

### 3.2. Experimental Settings

The BERT model implementation was designed to comprise three transformer encoders, each of them characterized by a two-head attention mechanism. The size of the feed-forward neural network layers was set up to 256 neurons, while the embedding dimension was defined as 64. The training process relied on mini-batch stochastic training based on the cross-entropy cost function and Adam optimizer [52]. To measure the performance on newcoming data, we randomly allocated two portions of the dataset into a training set and a test set, including 80% and 20% of the users.

To deliver a clearer assessment, TraceBERT results were compared to traditional statistical approaches for modeling sequential data and transition probabilities. In particular, we reported three comparison baselines, each representing a different perspective of investigating the intrinsic motion characteristics of the dataset under study:

- Personal Markov model (PMM). It focuses on separately modeling individual movement patterns. Locations are represented as states and movements between locations as state transitions. Transition probabilities are estimated by counting each single user’s transitions between unmasked locations, therefore building, for each individual user, a “personal” transition matrix. At inference time, masked locations are predicted as the ones sharing the highest transition probability, according to the user-specific transition matrix, with their neighboring unmasked locations along the sequence.
- Global Markov model (GMM). It focuses on modeling collective movement patterns. Probability distributions are estimated by counting the collective state transitions of all users together, generating one global transition matrix. At inference time, masked locations are predicted as the ones sharing the highest transition probability, according to the global transition matrix, with their neighboring unmasked locations along the sequence.
- Global location co-visits (GLC). It focuses on grouping locations that are often visited together within the same trajectory segment, investigating the general shared relatedness between co-visited places. The predicted location of a given trace in the test set is identified as the one sharing the highest number of co-visits with the known locations in the trace, according to the global motion behavior observed in the training set. The sequential order is not modeled; only the overall amount of inherent co-visits, within the whole segment’s time span, is taken into account to generate the prediction.

### 3.3. Results

For an overall assessment of the model performance, we report the prediction results in the form of top-K accuracy metrics. If the real label is equal to one of the top K locations with the highest prediction probability, the accuracy is 1, otherwise it is 0; the global score refers to the average of all testing trajectories. Table 1 displays the comparison results. TraceBERT is shown to substantially outperform the baseline approaches, presenting a 6%, 11%, and 10% improvement, over the best baseline, in correspondence to the top-1, top-3, and top-5 accuracy scores, respectively.

As expected, PMM, solely modeling individual mobility, implies very low performances in this motion regime. GMM and GLC, which take into account the collective information of all users, present a significant improvement, with GMM exceeding GLC, meaning that neighboring location transition probabilities provide a better-focused information than a general estimation of location co-visits. TraceBERT, however, exhibits an additional increment, averagely overcoming the best baseline’s accuracy of 5.4 percentage points (with a peak of 6.75 points), therefore demonstrating its powerful capability of mining intrinsic trajectory patterns.

Additionally, we analyzed how the accuracy scores are affected by different motion characteristics, segmenting the performance evaluation according to different values of mobility features.

Table 2 reports the accuracy scores for several ranges of traveled distance within the 7 h trajectory segment. Five bins were considered: ≤10 km, 10–25 km, 25–50 km, 50–100 km, and ≥100 km. Observing the results, despite an expected overall lower performance in correspondence of longer traveled distances, PMM always behaves poorly, while GMM and GLC tend to decrease their performance as the distance increases. TraceBERT consistently exceeds every baseline in each distance bin, with a remarkable improvement for very long distances (≥100 km).

Table 3 shows, instead, the scores for several ranges of radius of gyration (ROG), according to the bins of ≤3 km, 3–10 km, 10–32 km, and ≥32 km. Reinforcing the previous statements, we notice a tendency of performance decrease for increasing ROG values, poor behavior of PMM, positive achievements of GMM and GLC towards small values (≤3 km) and a corresponding consistent drop in performance for very large values (≥32 km). TraceBERT, once again, overcomes the baselines, slightly exceeding the GMM scores in the ≤3 km bin, and progressively enlarging the difference as the ROG grows, greatly outperforming every method for very large ROG values (≥32 km).

**Figure 3:**
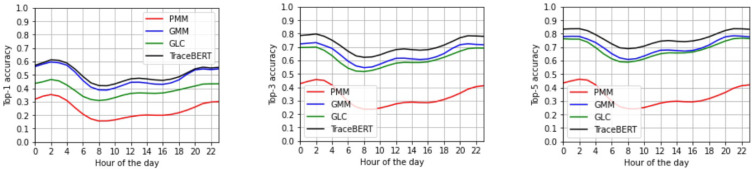
Top-1, top-3, and top-5 prediction accuracy scores (from **left** to **right**) over the 24 h of the day.

Moreover, we inspected the performance variation in different hours of the day. Figure 3 reports the top-K accuracies of each model over time. While the scores improve in the evening and nighttime because of the higher motion regularity, rush hours are reported to be easier to predict in the afternoon rather than in the morning. Nonetheless, TraceBERT was proved to overcome the other approaches in every hour, with a larger accuracy gap in correspondence of morning and afternoon hours, indeed when mobility becomes more chaotic.

Results were then investigated according to the amount of missing information, in terms of the number of masked locations along the trajectory segments. This represents a sign of evaluation measure for variable degrees of missing data gaps, ranging from moderate to severe information loss. Table 4 shows the top-K accuracies in correspondence of different numbers of masked locations per segment, namely 1–2 locations, 3–4 locations and over 5 locations. Besides a reasonable drop when increasing the missing location information level, the superiority of TraceBERT is once again clearly exhibited. However, despite it always exceeds every baseline, its performance seems to be more negatively affected by big information gaps, since the model cannot take full advantage of the implicit information derived from a wide context of known locations.

**Figure 4:**
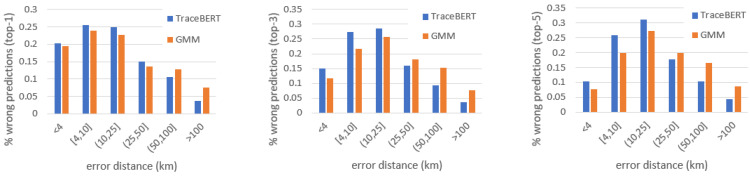
Bar graphs reporting the error distance distribution of the masked locations that are wrongly predicted by both TraceBERT and GMM (from **left** to **right**: wrong predictions in top-1, top-3, and top-5, respectively).

Finally, particular attention is directed to the analysis of prediction errors, targeting those cases when the model is not able to identify the correct missing locations. The outputs of TraceBERT were compared with GMM, the baseline with the best overall accuracy scores, to verify their error differences when both methods are mispredicting. Figure 4 depicts the bar graphs reporting the error distance distribution of the masked locations that are wrongly predicted by both approaches. The error distance of a top-K prediction is calculated as the minimum distance between the real target and each of the K predicted candidates. The figures suggest a clear trend of TraceBERT for prediction errors with a shorter error distance.

**Figure 5:**
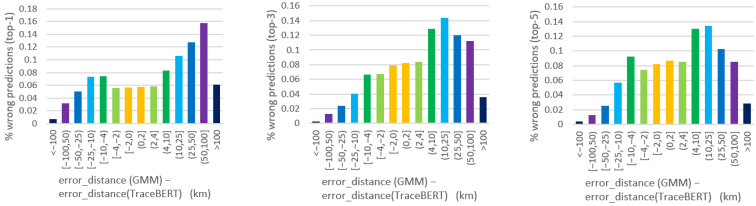
Bar graphs reporting the difference of error distance between GMM and TraceBERT in the case of common misprediction (from **left** to **right**: wrong predictions in top-1, top-3, and top-5, respectively).

Furthermore, if we analyze both models’ misprediction on the same corresponding masked location, we can directly derive their difference of error distance with regard to the same target gap. Figure 5 reports the subtraction $error\_distance\left(GMM\right)-error\_distance\left(TraceBERT\right)$. A positive value means that the wrong prediction provided by TraceBERT is closer in space to the real one, compared to the GMM solution; a negative value is instead in favor of the baseline. The high bars on the right side of the plots imply a substantial number of masked locations whereby GMM encounters prediction errors registering an error distance of a few tens of km larger than the TraceBERT case. Consequently, our approach, in addition to the better accuracy scores, also provides shorter error distances.

### 3.4. Discussion

We introduced a novel approach for reconstructing spatial–temporal gaps along incomplete trajectory segments. The methodology relied on historical collective large-scale human mobility and a deep learning-based model adapted to process location sequences. In particular, the NLP-related state-of-the-art BERT model was proposed in the context of trajectory analysis, evaluating its potential use within the human motion domain. We investigated the capabilities of this approach in the particular case of individual users with short data history and non-repetitive behaviors, whereby prediction algorithms approximating single probability distributions are not reliable.

The comparative evaluation highlighted indeed the problem of a probabilistic strategy based on a single individual’s mobility in this motion regime, indicating the necessity of collective motion information. This collection of non-repetitive trajectories leads the transition-probability-based Markov model to generally outperforming an approach fully based on location co-visits, highlighting the importance of location ordering and directionality. On the other hand, BERT, directed to mine complex patterns in sequential data, overcame the other approaches, indicating a prominent higher feasibility of identifying the correct missing locations along individual motion traces.

In addition, we examined how predictability was altered by various motion characteristics. Besides the reasonable trend of local movements to be more predictable than long-distance mobility (the first ones implicitly define a restricted set of potential candidates, whereas the second case imply a wider explored area including a larger amount of likely locations), our model was always reported to present higher accuracy scores than the baselines. Significantly, it exhibited the largest accuracy improvement exactly towards high values of traveled distance and ROG, therefore demonstrating a valuable predictability even for very wide explored areas. Furthermore, we provided an additional focus according to the time variable, organizing the accuracy scores with respect to the hour of the day. Again, TraceBERT outperformed the baselines, presenting a consistent improvement over the 24 h, with a higher predictability of afternoon hours over morning hours.

An additional investigation highlighted the performance on the basis of the amount of missing spatial–temporal information, registering prediction capabilities in correspondence of different numbers of masked locations along the sequences. TraceBERT, once again, exceeded the baselines, achieving promising results even for very fragmented location sequences. The largest improvement, however, was obtained in correspondence of those traces with a smaller amount of missing information, as the neural network could fruitfully take advantage of a more complete context surrounding the missing locations and therefore acquire a better hint to correctly define their reconstruction.

A final important perspective was then related to the evaluation of the prediction error. While the best possible solution would be the correct detection of a missing location, whenever a misprediction occurs, it would be still valuable to assess the entity of this mistake. Comparisons revealed that the error distance of TraceBERT was often a few tens of km smaller than the best baseline approach. This underlined an inherent tendency of making less serious prediction errors, hence reinforcing its superiority even more.

In conclusion, we assessed the feasibility of converting the NLP-oriented BERT approach for masked language modeling into an explicit deep learning model for processing location-based trajectories, therefore introducing its use in the human mobility domain. Although the main purpose was strictly methodological, our research opens to a wide variety of possible applications dealing with location-based services. The most straightforward use would be aimed to improve the data quality for subsequent downstream tasks, generating complete trajectories out of sparse recording observations or missing recording gaps. Such newly generated traces can be then utilized in a multitude of implementations involving trajectory data. Among many options, correctly reconstructing missing mobility information of single individuals can lead to potential improvements in the quality of personalized recommendations and touristic experiences, assuming that the previously visited venues and attractions intrinsically carry information on the user’s characteristics and potential future trips, further leading to promotions, service opportunities and demand estimations. In broader terms, the reconstruction of individual mobility traces can improve the overall view of the evolving time-dependent human collective distribution over the territory.

From a higher-level perspective, this work contributes to the investigation of the potential of advanced deep neural network methodologies on human motion studies, proposing a feasible adaptation of the BERT model as a promising tool for trajectory pattern mining.

## 4. Conclusions

Inspired by advances in computational linguistics, this paper explored the possibility of converting cutting-edge language modeling methodologies into the human mobility domain. In particular, we proposed a deep learning approach for inferring missing location gaps along incomplete trajectory segments. Trained on people’s collective mobility, the model was able to automatically detect motion patterns from location sequences in a purely data-driven manner. While the methodology is in principle applicable to any kind of trajectory-related context, we selected a use case inserted in the field of tourism mobility, naturally characterized by short and non-repetitive motion traces, aiming to reconstruct short-term foreign tourists’ motion activity.

The workflow comprised four parts, i.e., the pre-processing of raw traces into fixed-step location sequences; a random location-masking procedure based on a selected masking probability; the training of a BERT-like neural network, including transformer architectures with attention mechanism; and the final inference phase on incomplete testing trajectories. Our proposed approach has been shown to outperform baseline methodologies, denoting a remarkable potential for detecting complex mobility patterns. We believe that our findings will inspire further research activities on the application of sequence-oriented advanced neural network models towards human mobility analysis.

Future extensions of our work may point to multiple directions. An option would be to explore possible augmentations of trajectory data with further information, such as tourists’ personal characteristics or explicit time features. A different research direction may focus instead on trajectory reconstruction at a smaller scale, investigating more detailed resolutions in time and space. In addition, a variety of diverse mobility-based use cases may be tackled, even exploring more sophisticated implementations of technical aspects such as further masking strategies or variable-length trajectory segments. Finally, a more conceptual direction should target a better theoretical clarification on the inherent semantic choices of the model with regard to location dependencies, digging into the causes that lead to filling a certain location gap in a certain specific way.

In conclusion, the adaptation of the BERT architecture for reconstructing trajectory segments represents a promising tool in the field of motion analysis, deserving further attention and exploratory studies to more deeply investigate its potential in a range of applications within the mobility domain.

## Figures and Tables

**Table 1:** Overall accuracy comparison between TraceBERT and the three baseline approaches, namely the personal Markov model (PMM), global Markov model (GMM), and global location co-visits (GLC).

	Top-1 Accuracy	Top-3 Accuracy	Top-5 Accuracy
PMM	0.2050	0.2931	0.3012
GMM	0.4482	0.6198	0.6817
GLC	0.3658	0.5890	0.6613
TraceBERT	0.4745	0.6870	0.7492

**Table 2:** Comparison of top-1 accuracy, top-3 accuracy (in round brackets), and top-5 accuracy (in square brackets) for different ranges of traveled distance.

Traveled Distance =	≤10 km	10–25 km	25–50 km	50–100 km	≥100 km
PMM	0.4005 (0.5391) [0.5414]	0.2644 (0.4027) [0.4153]	0.1829 (0.2784) [0.2938]	0.1321 (0.1931) [0.2040]	0.0622 (0.0827) [0.0855]
GMM	0.7039 (0.9227) [0.9582]	0.5449 (0.7770) [0.8523]	0.4458 (0.6273) [0.7143]	0.3627 (0.5101) [0.5829]	0.2237 (0.3191) [0.3686]
GLC	0.5864 (0.9070) [0.9550]	0.4642 (0.7648) [0.8559]	0.3635 (0.6084) [0.7135]	0.2903 (0.4791) [0.5659]	0.1617 (0.2545) [0.3014]
TraceBERT	0.7145 (0.9524) [0.9741]	0.5604 (0.8441) [0.9069]	0.4671 (0.7041) [0.7892]	0.3916 (0.5792) [0.6586]	0.2722 (0.4085) [0.4769]

**Table 3:** Comparison of top-1 accuracy, top-3 accuracy (in round brackets), and top-5 accuracy (in square brackets) for different ranges of the radius of gyration.

ROG =	≤3 km	3–10 km	10–32 km	≥32 km
PMM	0.3531 (0.5059) [0.5117]	0.2190 (0.3279) [0.3428]	0.1634 (0.2249) [0.2343]	0.0705 (0.0908) [0.0931]
GMM	0.6305 (0.8958) [0.9449]	0.4885 (0.6709) [0.7598]	0.4184 (0.5510) [0.6116]	0.2401 (0.3347) [0.3823]
GLC	0.5603 (0.8961) [0.9495]	0.3981 (0.6649) [0.7722]	0.3152 (0.4980) [0.5804]	0.1709 (0.2637) [0.3099]
TraceBERT	0.6509 (0.9360) [0.9679]	0.4995 (0.7464) [0.8301]	0.4430 (0.6139) [0.6810]	0.2903 (0.4250) [0.4915]

**Table 4:** Comparison of top-1 accuracy, top-3 accuracy (in round brackets), and top-5 accuracy (in square brackets) for different amounts of masked locations per segment.

# Masked Locations =	1–2 Locations	3–4 Locations	≥5 Locations
PMM	0.2732 (0.4043) [0.4201]	0.1719 (0.2365) [0.2394]	0.0370 0.0373 0.0374
GMM	0.4670 (0.6357) [0.6965]	0.4426 (0.6155) [0.6778]	0.3765 (0.5562) [0.6212]
GLC	0.3691 (0.5977) [0.6722]	0.3654 (0.5871) [0.6584]	0.3495 (0.5505) [0.6166]
TraceBERT	0.5017 (0.7177) [0.7789]	0.4640 (0.6760) [0.7386]	0.3875 (0.5830) [0.6471]

## Data Availability

Not applicable.
